# The Epac1 Protein: Pharmacological Modulators, Cardiac Signalosome and Pathophysiology

**DOI:** 10.3390/cells8121543

**Published:** 2019-11-29

**Authors:** Marion Bouvet, Jean-Paul Blondeau, Frank Lezoualc’h

**Affiliations:** 1 INSERM UMR-1048, Institut des Maladies Métaboliques et Cardiovasculaires, 31432 Toulouse, France; marion.bouvet@inserm.fr; 2 Université de Toulouse - Paul Sabatier, 31432 Toulouse, France; 3 Faculté de Pharmacie, Université Paris-Sud, 92296 Châtenay-Malabry CEDEX, France; jean-paul.blondeau@u-psud.fr

**Keywords:** 3′,5′-cyclic adenosine monophosphate (cAMP), compartmentalization, cardiac disease, exchange protein directly activated by cAMP 1 (Epac), small molecules

## Abstract

The second messenger 3′,5′-cyclic adenosine monophosphate (cAMP) is one of the most important signalling molecules in the heart as it regulates many physiological and pathophysiological processes. In addition to the classical protein kinase A (PKA) signalling route, the exchange proteins directly activated by cAMP (Epac) mediate the intracellular functions of cAMP and are now emerging as a new key cAMP effector in cardiac pathophysiology. In this review, we provide a perspective on recent advances in the discovery of new chemical entities targeting the Epac1 isoform and illustrate their use to study the Epac1 signalosome and functional characterisation in cardiac cells. We summarize the role of Epac1 in different subcompartments of the cardiomyocyte and discuss how cAMP–Epac1 specific signalling networks may contribute to the development of cardiac diseases. We also highlight ongoing work on the therapeutic potential of Epac1-selective small molecules for the treatment of cardiac disorders.

## 1. Introduction

**Figure 1:**
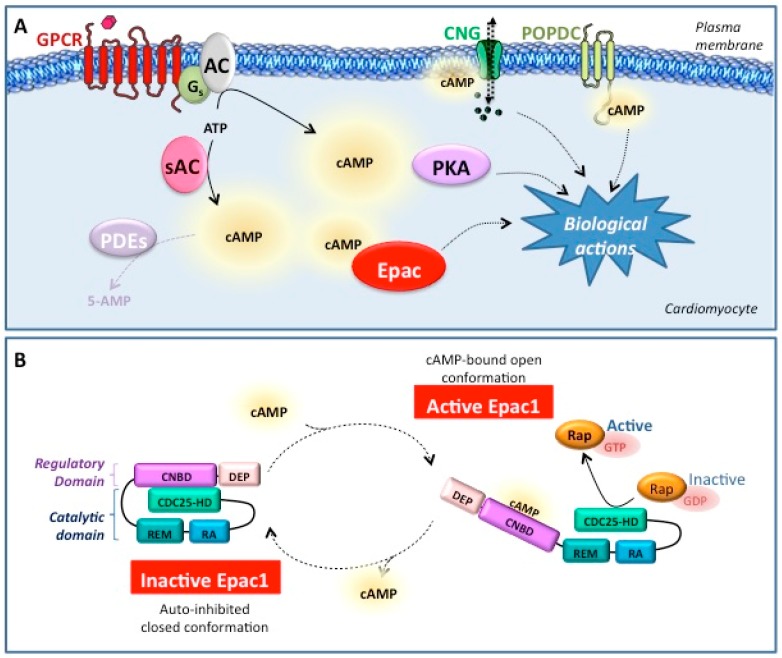
3′,5′-Cyclic adenosine monophosphate (cAMP)-mediated signalling and exchange protein directly activated by cAMP (Epac)1 activation. (**A**) cAMP effectors in cardiac myocytes. Intracellular pool of cAMP is generated from ATP by two classes of adenylyl cyclases (AC): the membrane-bound AC and soluble AC (sAC). While membrane-bound AC is activated in response to Gαs-coupled G protein–coupled receptors (GPCRs) stimulation, sAC is directly activated by Ca^2+^ and bicarbonate. Phosphodiesterases (PDE), that hydrolyse cAMP to 5′-AMP, limit the local concentration of cAMP and sculpt the cAMP gradient. The biological effects of cAMP are ensured by four downstream effectors: protein kinase A (PKA), exchange proteins directly activated by cAMP proteins (Epac), cyclic nucleotide gated (CNG) ion channels, and Popeye domain-containing (POPDC) proteins. (**B**) Mechanism of Epac1 activation. The catalytic region of Epac1 contains the cell division cycle 25 homology domain (CDC25-HD), Ras-association (RA) domain and Ras-exchange motif (REM) domain. The Epac1 regulatory region includes the cyclic-nucleotide-binding domain (CNBD) and Dishevelled, Egl-10, Pleckstrin domain (DEP). Binding of cAMP to the CNBD induces a conformational change that opens the catalytic CDC25-HD domain from autoinhibitory restraints and thereby permits GTP-loading of Rap.

3′,5′-Cyclic adenosine monophosphate (cAMP) is a universal second messenger that regulates a multitude of physiological and pathological processes. Synthesis of cAMP from adenosine triphosphate (ATP) is generated by membrane-bound adenylyl cyclases (AC) upon activation of Gs-coupled G protein-coupled receptors [1]. Soluble adenylyl cyclase (sAC) constitutes the second source of cAMP and is activated by bicarbonate and Ca^2+^ [2]. Intracellular levels and diffusion of cAMP are further regulated through hydrolysis to 5′AMP by a large family of cAMP phosphodiesterases (PDEs) [3]. The biological effects of cAMP are ensured by four main downstream effectors: the protein kinase A (PKA), exchange proteins directly activated by cAMP proteins (Epac), cyclic nucleotide gated (CNG) ion channels, and Popeye domain-containing (POPDC) proteins [4,5,6,7] (Figure 1A). In addition, the molecular scaffolds, A-kinase anchoring proteins (AKAPs) sequester cAMP effectors and PDEs into discrete subcellular compartments to maintain localized pools of cAMP and control therefore the specificity and cellular actions of this second messenger [8]. This compartmentalization permits the differential activation of cAMP effectors. Current evidence indicate that aberrant cAMP signalling through dysregulation of cAMP compartmentalization contributes to pathological cardiac remodelling and heart failure (HF), a major cause of death world-wide [9]. However, the molecular mechanisms by which altered cAMP signalling promotes HF development are not fully understood.

Classically, most biologic effects of cAMP in cardiomyoctes have been assigned to PKA, which promotes the acute effect of the β-adrenergic receptor (β-AR) on cardiac contractility through phosphorylation of L-type calcium channels, sarcoplasmic ryanodine receptors (RyR), and the sarcoplasmic reticulum Ca^2+^-ATPase 2a regulator protein phospholamban [10]. β-AR activation is an integral part of the cardiac fight-or-flight response, however, its chronic activation as observed in the context of HF contributes to pathological cardiac remodelling, contractile dysfunction, and arrhythmia [11]. The detrimental effects of sustained β-AR stimulation are in line with the observation that plasma norepinephrine concentration in HF patients correlates with the degree of cardiac dysfunction and mortality [12]. 

The cAMP-binding proteins, Epac1 and Epac2 and their myocardial expression were identified two decades ago, raising the question of their functional role and disease implication in the heart [9,13,14]. Epac proteins largely function in a PKA-independent manner and are a novel means for regulating the specificity of cAMP signalling. The following sections will describe the Epac1 protein and Epac1 pharmacological tools including cAMP analogues and Epac1 antagonists. We summarize recent key findings regarding the Epac1 signalosome and functions that can lead to a better understanding of its pathophysiological role in the heart. We also highlight the potential benefits of targeting Epac1 proteins for the treatment of cardiac diseases.

## 2. Epac1 Protein

### 2.1. Epac Genes and Transcripts

Epac proteins are guanine-nucleotide-exchange factors (GEFs) and were discovered 20 years ago by two independent research groups as new downstream effectors of cAMP that mediate the PKA-independent activation of small G-proteins of the Ras superfamily, Rap1 and Rap2 [13,14]. The Epac proteins, Epac1 and Epac2 are encoded by two distinct genes, which give rise to various transcripts [7]. In humans, Epac1 is encoded by the RAPGEF3 gene that produces three transcripts whose functional differences are unknown [15]. Transcript variant 1 encodes the full length Epac1A protein with 923 amino acids (AA) while the transcript variants 2 and 3 produce the same and shorter Epac1B isoform of 881 AA length with a shorter N-terminus compared to Epac1A (https://www.ncbi.nlm.nih.gov/gene/10411). The human and mouse Epac2 genes, RAPGEF4 have currently five different validated transcript variants in the NCBI database. Four of which have been described in the literature (Epac2A1, Epac2A2, Epac2B, and Epac2C) [7]. While Epac2A corresponds to the longest Epac2 isoform (1011 AA), the other transcript variants produce shorter Epac2 isoforms (993, 867, 791) that stem from variations in the transcriptional start site and alternative splicing [7,16]. While Epac1 mRNA is nearly ubiquitously expressed with high expression levels in the human kidney and heart, the Epac2 isoforms exhibit a more restricted expression pattern and are mainly expressed in the brain and endocrine tissues [7,13,14]. 

### 2.2. Epac1 Structure

Epac1 and Epac2 are multidomain proteins and share similar structural organization [4,7,9]. Both Epac2 and Epac1 contain two main parts: an N-terminal regulatory region and a C-terminal catalytic domain (Figure 1B). The N-terminal part of Epac1 and Epac2 functions as an auto-inhibitory domain that inhibits Epac catalytic activity in the absence of cAMP. The N-terminal part of Epac1 contains a Dishevelled, Egl-10, Pleckstrin (DEP) domain and a cyclic-nucleotide-binding domain (CNBD), which are involved in Epac1 localization and activation, respectively (Figure 1B). The longer isoform of Epac2 (Epac2A) contains an additional CNB domain (CNBD-A), which binds cAMP with low affinity, is not required for Epac2 activation by cAMP, and is involved in the subcellular localization of Epac2A. CNBD is a conserved domain found in the regulatory subunit of mammalian proteins that respond to cAMP such as PKA and CNG ion channels. It sterically blocks the access of Rap proteins to the catalytic site of Epac. Binding of cAMP to Epac induces huge conformational changes within the protein and releases the auto-inhibitory effect of the N-terminus of the protein, leading to Rap1 activation [17] (Figure 1B). 

The C-terminal catalytic region of Epac1 consists of three principal domains. The Ras-exchange motif (REM) domain is involved in the stabilization of the active conformation of Epac [18]. The Ras-association (RA) domain is a protein interaction motif and the cell division cycle 25 homology domain (CDC25-HD) promotes the exchange of GDP for GTP on Rap GTPases (Figure 1B). Of note, Epac1 can undergo posttranslational modifications such as phosphorylation that can fine-tune its activity. This is well illustrated with G-protein receptor kinase 2 (GRK2)-dependent phosphorylation of Epac1 which inhibits the translocation of Epac1 to the plasma membrane and thereby Rap1 activation [19]. Other putative phosphorylation sites are present in Epac1 but their functional relevance needs to be studied.

## 3. Small-Molecule Epac1 Modulators

The growing evidence that Epac proteins are involved in a large number of major pathophysiological processes in various tissues stimulated the search for Epac-selective pharmacological modulators (agonists and inhibitors). These compounds are expected not to interfere with the cAMP/PKA-dependent pathway, and, optimally, also discriminate between the two Epac isoforms, Epac1 and Epac2.

### 3.1. Epac1 Agonists

#### 3.1.1. Cyclic Nucleotides 

The cAMP analogue 8-(4-chlorophenylthio)-2′-O-methyladenosine-3′,5′-cyclic monophosphate, in which a para-chlorophenylthio (pCPT) substituent is present at the 8-position of cAMP and a 2′-O-methyl group substituted the ribose 2′-hydroxy group, was developed through rational drug design [20]. This compound is abbreviated as 8-CPT-cAMP or 8-pCPT-cAMP, and also frequently referred to as “007”. It is ≈15 times more efficient than cAMP to activate Epac1 in vitro, and ≈30–100 times less potent than cAMP as an activator of type-I and type-II holoenzymes of PKA [20]. 

Furthermore, 8-pCPT-cAMP was also reported to preferentially activate Epac1 over Epac2 in vitro [21]. Determination of agonist effective concentrations which produced 50% of the maximum response (EC50), in a functional fluorescence-based Rap1 GEF assay, indicated that 8-pCPT-cAMP was six times more potent than cAMP to activate Epac1 at half-saturation, whereas the efficacy of 8-pCPT-cAMP to activate Epac2 was 50% lower than that of cAMP. Assessment of the maximal GEF activities at saturating concentrations of agonists (Vmax) resulted in the same pattern, 8-pCPT-cAMP behaving as a “super agonist” for Epac1 and behaving as a partial agonist for Epac2. The preferential activation of Epac1 by 8-pCPT-cAMP compared to Epac2 was confirmed in a later independent study [22]. In addition, the authors, using site-directed mutagenesis and activity assays, demonstrated that a single AA determines the differential response of Epac1 and Epac2 to 8-pCPT-cAMP, namely Epac1 Gln270 corresponding to Epac2 Lys40.

Several 8-pCPT-cAMP derivatives were used in order to facilitate Epac activation in intact cells and to avoid degradation by intracellular phosphodiesterases (PDEs). These include a phosphorothioate derivative of 8-pCPT-cAMP (Sp-8-pCPT-2’-O-Me-cAMPS), which is resistant to PDE hydrolysis [23], and an acetoxymethyl ester form of 8-pCPT-cAMP (8-pCPT-2′-O-Me-cAMP-AM), which readily crosses the plasma membrane and is hydrolyzed by cellular esterases to release the biologically active parent compound [24].

A considerable number of studies used 8-pCPT-cAMP and its derivatives to unravel Epac functions in various cell types including cardiomyocytes [7,9] (see below). However, 8-pCPT-cAMP and its nonhydrolysable phosphorothioate derivative are inhibitors of several PDEs, which can result indirectly in an increase in cAMP or cGMP intracellular levels and activation of cAMP/PKA or cGMP/protein kinase G (PKG) pathways [25]. Therefore cAMP analogues that are considered as being specific Epac activators interact with several off-targets, pointing to the requirement of control experiments to ascertain that the observed effects of these analogues are indeed acting via Epac proteins [26]. The search for non-nucleotide small molecules with Epac agonist properties was stimulated owing to difficulties in identifying cyclic nucleotides analogues with optimal pharmacokinetics, agonist properties specific to each Epac isoform, and reduced off-target effects.

#### 3.1.2. Non-Cyclic Nucleotide Small Molecules 

Sulfonylureas, which are drugs widely used for the treatment of type 2 diabetes mellitus, were reported to be selective activators of Epac2 [27]. However, subsequent studies showed that the sulfonylureas glibenclamide and tolbutamide (Table 1) were unable to compete with the fluorescent cAMP analogue 8-NBD-cAMP (8-(2-[7-Nitro-4-benzofurazanyl]aminoethyl-thio) adenosine-3′, 5′-cyclic monophosphate) for binding to Epac2, as well as to stimulate the Epac2-mediated Rap1 GEF assay in vitro [28]. In contrast, tolbutamide selectively activated an Epac1-based FRET sensor in whole cells [29], suggesting that this sulfonylurea is in fact able to promote Epac1 activation in cells, independently of PKA activation. Unfortunately, millimolar concentrations of tolbutamide were necessary to observe a significant activation of Epac1.

The fluorescent cAMP analogue 8-NBD-cAMP was used as a ligand of the isolated CNBDs of Epac1 and 2 to screen for binding competitors [30]. Compounds, named I942 and I178, were found to inhibit 8-NBD-cAMP binding, suggesting interaction with Epac CNBDs. In a functional fluorescence-based Rap1 GEF assay, I942 compound (Table 1) showed partial agonist activity towards Epac1 but not Epac2 or PKA, with an EC50 = 50 μM and a maximal potency of less than 10% of that of cAMP [30]. I942 was reported to promote Epac1-Rap activation in HEK293T cells stably expressing Epac1, and to induce SOCS3 expression and suppress Epac1-dependant IL6-stimulated JAK/STAT3 signalling in cultured vascular endothelial cells [31]. It may be noted that I942 shares a common chemical substructure (N-formyl-methylbenzenesulfonamide) with tolbutamide, whose Epac1-activating effect is discussed above. Although I942 promoted Epac1-Rap activation in the absence of cAMP, it behaved as a competitive inhibitor of cAMP-induced Epac1 activation [30], suggesting a complex pharmacological behaviour and pointing out the need for chemical optimization of this class of compounds.

### 3.2. Epac1 Competitive Inhibitors

Efforts have been undertaken to identify non-cyclic nucleotide small molecules able to selectively inhibit Epac activities without affecting PKA activity. To this end, a prototypical high-throughput screening assay was developed for identifying compounds that directly compete for binding of the fluorescent cAMP derivative 8-NBD-cAMP to full-length recombinant Epac2 [32,33]. One hit, ESI-08 (Table 1), inhibited both cAMP-stimulated Epac1 and Epac2 activity [33]. Structural modifications of ESI-08 and structure-activity relationship (SAR) studies led to the identification of a more potent compound, HJC0197 (Table 1), which blocked Epac1 and Epac2 GEF activities at 25 µM in the presence of equal molar concentration of cAMP, and had no effect on PKA activity. Another hit compound, ESI-09 (Table 1), was identified, which was also found to be nonselective to Epac1 and Epac2, inhibiting cAMP-stimulated GEF activities of both isoforms in the µM range with similar IC50s [34].

It should be noted that ESI-09 and HJC0197 were reported to act as chemicals with general protein denaturing properties, raising concerns about their specificity as Epac inhibitors [35]. Some Epac small-molecule inhibitors, including ESI-09, are prone to aggregation-based inhibition leading to false positives arising from nonspecific binding. The mechanisms by which additives such as Triton X-100 and serum albumin can attenuate this adverse effect have been studied in detail by combination of biophysical techniques [36]. Biochemical characterization and SAR studies indeed suggested that ESI-09 inhibited activity of both Epac1 and Epac2 at concentrations well below those that induce protein denaturation [37]. A number of works were published using ESI-09 or its derivatives, helping to establish the ex vivo or in vivo involvement of Epac1 and/or Epac2 in various cAMP-dependent pathways [7].

Chemical optimization work [38] based on substitution for the phenyl ring of ESI-09 produced several compounds such as HJC0726 and NY0123 (Table 1), which equally inhibited Epac1 GEF activity with IC50 value of 2.4 μM. However, these compounds, particularly NY0123, were even better inhibitors of 8-NBD-cAMP binding to Epac2 than to Epac1. Recently, further chemical optimizations involving modifications of the isoxazole and phenyl rings of ESI-09 have resulted in the discovery of several novel Epac antagonists, among which NY0460 and NY0562 (Table 1) show low micromolar inhibitory activities (IC50 = 2.4 µM and 2.7 µM, respectively), but without specificity for Epac1 with respect to Epac2 [39]. In another optimization study, ZL0524 (Table 1) compound has been discovered as a potent Epac inhibitor with IC50 values of 3.6 µM and 1.2 µM against Epac1 and Epac2, respectively [40], and therefore without specificity in favour of Epac1.

In conclusion, there is a range of competitive Epac pan-inhibitors with high affinity that do not significantly affect the activity of other proteins involved in cAMP signalling, particularly PKA. Several of these pharmacological tools are commercially available, and have been used to study the involvement of Epac proteins in various pathophysiological processes. However, to our knowledge, there are no competitive Epac1 inhibitors at this stage that can directly antagonize Epac1 without simultaneously inhibiting Epac2. In order to ascertain that the observed effects of these dual-specificity inhibitors are indeed acting via Epac1, control experiments should be required involving Epac1 gene deletion and/or use of Epac2-specific competitive antagonists such as ESI-05 [41] or HJC0350 compounds [42].

### 3.3. Epac1 Noncompetitive Inhibitors

#### 3.3.1. Compound 5376753

Virtual screening of a Chembridge library using a 3D model of the apo-Epac1 structure in its inactive state has led to the identification of a barbituric acid derivative, compound 5225554. This compound could be docked to a putative allosteric site located near the hinge region of Epac1, and thus outside the cAMP-binding site [43]. 5225554 inhibited forskolin-stimulated Rap1 activation but had no effect on PKA activity [43].

Compound 5376753 (Table 1), a thiobarbituric acid analogue of 5225554, was a more effective Epac inhibitor than the parent compound [44]. 5376753 inhibited Rap1 activation in cells with IC50 = 4 μM. 5376753 behaved as a noncompetitive allosteric inhibitor of cAMP-stimulated Epac1 in the Camyel Bioluminescence Resonance Energy Transfer (BRET)-based assay, reducing maximal activation of Camyel, while the apparent affinity for cAMP remained unchanged. Rap1 activation was also inhibited by 5376753 in cells overexpressing Epac2, showing that 5376753 is a dual Epac1 and Epac2 inhibitor. 5376753 inhibited a biological function mediated by Epac, namely the cAMP-induced migration of adult rat cardiac fibroblasts in primary culture [44]. 

#### 3.3.2. Compound AM-001

A thieno(2,3-b)pyridine derivative (Table 1), named AM-001, was identified by screening a diverse in-house chemical collection using an in vitro assay based on a Camyel Epac1-BRET biosensor [45]. AM-001 decreased the cAMP-induced maximal BRET response in a concentration dependent-manner (IC50 = 50 µM) but did not affect the EC50 of cAMP, a kinetic feature of noncompetitive inhibition. AM-001 inhibited in vitro Epac1 GEF activity with an IC50 = 48 µM, independent of the agonist concentration used to activate Epac1, thus confirming the noncompetitive behaviour of AM-001. In contrast to its antagonism to Epac1, AM-001 was ineffective in suppressing Epac2 GEF activity. Furthermore, it also did not influence type I/II PKA holoenzyme activities. In addition to the use of AM-001 as a molecular tool to study signaling pathways that are specifically regulated by Epac1, potential therapeutic applications were suggested by experiments performed in vitro and in vivo [45] (see Section 4 below).

### 3.4. Epac1 Uncompetitive Inhibitors

Brefeldin A is a known uncompetitive inhibitor of a GEF for adenosine diphosphate-ribosylation factor (Arf). Brefeldin A was also reported to antagonize 8-pCPT-cAMP action on synaptic transmission in vivo, suggesting that this compound is an inhibitor of Epac action [46]. Nevertheless, no evidence of a direct effect of brefeldin A, up to 100 mM, could be demonstrated on the in vitro Epac2 and Epac1 exchange reaction [28,35].

A fluorescence-based Rap1 exchange assay was used to identify Epac inhibitor compounds from the “Chimiothèque Nationale Essentielle” compound library [47]. A tetrahydroquinoline analogue, named CE3F4 (Table 1), inhibited Epac1 GEF activity in vitro, without directly disrupting the interaction between Epac1 and Rap1, or competing for binding of cAMP and other cyclic-nucleotide agonists to Epac1. CE3F4 also did not influence protein kinase A holoenzyme activity in vitro.

**Table 1:** Non-nucleotide Epac1 modulators. (unk): unknown IC50 (no available dose-response); (O): orthosteric agonism; (C): orthosteric competitive inhibition; (NC): allosteric noncompetitive inhibition; (UC): allosteric uncompetitive inhibition.

Usual Name	IUPAC Chemical Name	Chemical Structure	Targeted Isoform	EC/IC50 (µM)	Mechanism	Ref.
AGONISTS						
Tolbu- tamide	N-(butylcarbamoyl)-4- methylbenzenesulfonamide	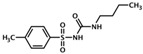	Epac1	> 2000	O	[27]
I942	N-[(2,4-dimethylphenyl)sulfonyl]-2-(2- naphthyloxy)acetamide	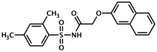	Epac1	50	O	[30]
ANTAGONISTS						
ESI-08	4-cyclohexyl-2-(2,5- dimethylbenzylthio)-6-oxo-1,6- dihydropyrimidine-5-carbonitrile	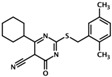	Epac1 & Epac2	unk	C	[33]
HJC0197	4-cyclopentyl-2-(2,5- dimethylbenzylsulfanyl)-6-oxo-1,6 dihydropyrimidine-5-carbonitrile	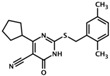	Epac1 & Epac2	unk	C	[33]
ESI-09	3-(5-tert-butylisoxazol-3-yl)-2-[(3- chlorophenyl)hydrazono]-3- oxopropionitrile	* 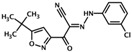 *	Epac1 & Epac2	3.2	C	[34]
HJC0726	2-(5-(tert-butyl)isoxazol-3-yl)-N-(3,5- dichlorophenyl)-2-oxoacetohydrazonoyl cyanide	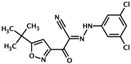	Epac1 & Epac2	2.4	C	[38]
NY0123	2-(5-(tert-butyl)isoxazol-3-yl)-2-oxo-N- (3,4,5-trichlorophenyl) acetohydrazonoyl cyanide	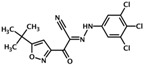	Epac1 & Epac2	2.4	C	[38]
NY0460	N-(3-trifluoromethyl-4-chlorophenyl)-2- oxo-2-(5-phenylisoxazol-3- yl)acetohydrazonoyl cyanide	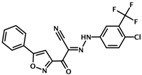	Epac1 & Epac2	2.4	C	[39]
NY0562	2-(benzo[d]isoxazol-3-yl)-N-(4-chloro- 3-(trifluoromethyl)phenyl)-2- oxoacetohydrazonoyl cyanide	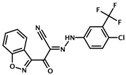	Epac1 & Epac2	2.7	C	[39]
ZL0524	(E)-N-(3,5-dichlorophenyl)-2-oxo-2- (5,6,7,8-tetrahydronaphthalen-2- yl)acetohydrazonoyl cyanide	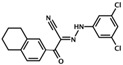	Epac1 & Epac2	3.6	C	[40]
5376753	5-{[5-(2,4-dichlorophenyl)-2- furyl]methylene}-2-thioxodihydro- 4,6(1H,5H)-pyrimidinedione	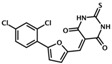	Epac1 & Epac2	4	NC	[44]
AM-001	3-amino-N-(4-fluorophenyl)-4-phenyl-6- (2-thienyl)thieno[2,3-b]pyridine-2- carboxamide	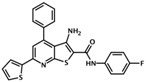	Epac1	48	NC	[45]
(R)-CE3F4	(2R)-5,7-dibromo-6-fluoro-2-methyl-1,2, 3,4-tetrahydroquinoline-1-carbaldehyde	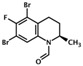	Epac1	4.2	UC	[21,47]

CE3F4 decreased both the maximal velocity of GDP exchange and the EC50 for cyclic nucleotide agonists, a kinetic feature of uncompetitive inhibition with respect to the cyclic nucleotide agonist. This is in agreement with the observed absence of effect of CE3F4 on the constitutive GEF activity of Epac (i.e., low intrinsic activity in the absence of an agonist) and the decrease of CE3F4 IC50 when increasing agonist concentration. CE3F4 therefore promoted a nonclassical type of uncompetitive enzyme inhibition, where the inhibitor (CE3F4) is uncompetitive with respect to the allosteric agonist (cAMP) rather than to the substrate (Rap1).

CE3F4 is a racemic mixture of two enantiomers, (R)-CE3F4 and (S)-CE3F4, owing to the presence of an asymmetric carbon atom in position 2 of the molecule (Table 1). It was found that (R)-CE3F4 inhibited Epac1 GEF activity with an IC50 (≈6 µM) which was ≈two-fold smaller than that of racemic CE3F4, and ≈10-fold smaller that of (S)-CE3F4 [21]. Moreover, (R)-CE3F4 displayed strong Epac isoform preference, being a 10-fold more potent Epac1 antagonist than (S)-CE3F4.

The uncompetitive mechanism of action of (R)-CE3F4 was partly deciphered using a combination of protein- and ligand-based NMR experiments [48]. It was established that (R)-CE3F4 binds within the Epac1 cAMP-binding domain (CNBD) at a subdomain interface distinct from the cAMP binding site, resulting in the formation of a cAMP:Epac1CNBD:(R)-CE3F4 ternary complex. (R)-CE3F4 acts as a wedge that stabilizes a cAMP-bound mixed-intermediate, which includes features of both the apo/inactive and cAMP-bound/active states of Epac1. The access of the Rap1 substrate to the catalytic domain is then prevented, as in the apo/inactive state. A single Epac1 versus Epac2 AA difference (Q270 in Epac1, corresponding to K405 in Epac2) was shown to account for the isoform selectivity of (R)-CE3F4 in favour of Epac1.

CE3F4 was successfully used in various studies to assess Epac1 biological action in cardiomyocytes (see Section 4 below) and other different cell types.

### 3.5. Conclusion on Epac1 Inhibitors

The efficacy of a competitive inhibitor towards a target protein—an enzyme or a receptor—decreases when the enzyme substrate (or receptor agonist) concentration increases. If the substrate (or agonist) concentration is greater than the Km (or Kd) of the target protein, the competitive inhibitor is expected to have a much higher affinity for the target protein, or be present at a high concentration, to be effective. On the other hand, a noncompetitive inhibitor which binds at an allosteric site both to the target protein-substrate (or target protein-agonist) complex and to the free target protein, will be more effective than a competitive inhibitor in the presence of an elevated concentration of substrate (or agonist) [49]. In the case of an uncompetitive inhibitor, which binds to an allosteric site of the target protein in complex with its substrate (or agonist) but not to the free enzyme, the higher the substrate (or agonist) concentration, the more effective the uncompetitive inhibitor is, which is the exact opposite of what happens with a competitive inhibitor. A competitive inhibitor will lose potency as substrate (or agonist) concentration increases, whereas an uncompetitive inhibitor will be particularly powerful at high substrate (or agonist) concentration, even if its intrinsic affinity is relatively moderate. This can be put in parallel with the fact that local cAMP levels can reach high micromolar concentrations in pathophysiological situations such as chronic activation of β-AR pathway in cardiomyocytes leading to chronic HF [9] (see Section 4 below). Therefore, uncompetitive and noncompetitive inhibitors may be of critical interest in stressed conditions.

Otherwise, noncompetitive and uncompetitive allosteric types of inhibition are expected to reduce risks of potential cross-reactivity between Epac inhibitors and other target proteins sharing related cyclic nucleotide binding sites, as could be the case for PKA, CNG ion channels, or POPDC. They are also expected to exhibit specificity between the two Epac isoforms, as is the case for the uncompetitive inhibitor (R)-CE3F4 and the noncompetitive inhibitors AM-001 and 5376753. However, since allosteric site(s) might be less phylogenetically conserved than the orthosteric active site of a given protein, attention should be paid to the transposition in one species to another of results obtained in vitro or in vivo with allosteric inhibitors.

## 4. Role of Epac Proteins in Cardiac Diseases

### 4.1. Epac1 and Pathological Cardiac Remodelling Leading to HF

Pathological cardiac remodelling is a chronic maladaptive process, characterized by myocardial hypertrophy, fibrosis, and deterioration of cardiac performance. It is a consequence of interactions between adaptive modifications of cardiomyocytes and negative aspects of adaptation such as cardiomyocyte death and fibrosis [50]. Multiple factors including ischemia, hormones (e.g., cathecholamines), and mechanical stretch promote the development of cardiac remodelling. Ischemic and load-induced remodelling are the most commonly encountered situations that progress to HF. Ischemic remodelling appears after myocardial infarction (MI) whereas load-induced remodelling occurs during pressure and/or volume overload in the setting of hypertension, valvular dysfunction, and cardiomyopathy.

Interestingly, Epac1 expression was markedly increased in various experimental models of myocardial pathological remodelling such as chronic catecholamine infusion and pressure overload induced by thoracic aortic constriction [51,52,53]. Epac1 was also upregulated at the end-stage of non-ischemic or ischemic human HF [52,53]. In support of a role of Epac1 in the progression of cardiac hypertrophy, ectopic overexpression of Epac1 or direct activation of Epac1 with the cAMP analogue, 8-pCPT-cAMP induced various features of cardiac hypertrophy in rat neonatal cardiac myocytes, including myofibrillogenesis, increased cell surface area, protein synthesis, and expression of the atrial natriuretic peptide (ANF) [54]. Additionally, Epac1 induced rat adult cardiomyocyte hypertrophy in a cAMP-dependent but PKA-independent manner in response to β-AR activation [52].

Recently, genetic Epac knockout mouse models have been generated to test whether these deleterious effects of Epac occurred in vivo. Epac1- or Epac2-null mice did not influence cardiac function and Ca^2+^ handling in rest conditions [55,56]. Accordingly, Epac proteins did not seem to play a major role in the regulation of the excitation–contraction coupling in response to acute β-AR stimulation [55,57]. However, the situation is different when the animals are subjected to cardiac stress conditions. Indeed, Laurent and colleagues [56] reported that Epac1-deleted mice displayed less cardiac hypertrophy and fibrosis during chronic β-AR stimulation compared with the control littermate animals. It was suggested that the mechanism for this protection was through prevention of cAMP induced autophagy. In another mouse model of Epac1 knockout, animals were resistant to cardiac apoptosis and fibrosis induced by aortic banding [58]. Of particular importance, Epac1-deleted mice exhibited an improved cardiac function in stress condition such as chronic isoproterenol infusion [56,58]. In agreement with these studies, Epac1 has been shown to play an important role in adenylyl cyclases type 5-mediated cardiac dysfunction in response to adrenergic stimulation [59]. Consistent with the genetic data, pharmacological inhibition of Epac1, by the Epac1-selective inhibitor AM-001, ameliorated contractility and attenuated cardiac hypertrophy, inflammation induced by chronic β-AR stimulation with isoprenaline in C57BL/6 mice [45]. These results are also in line with the preserved contractile reserve (tested by dobutamine-induced increase in contractility) observed in Epac1- (and not Epac2-) deleted mice subjected to myocardial pressure overload [58].

### 4.2. Epigenetic Regulation of Epac1 During Cardiac Remodelling

**Figure 2:**
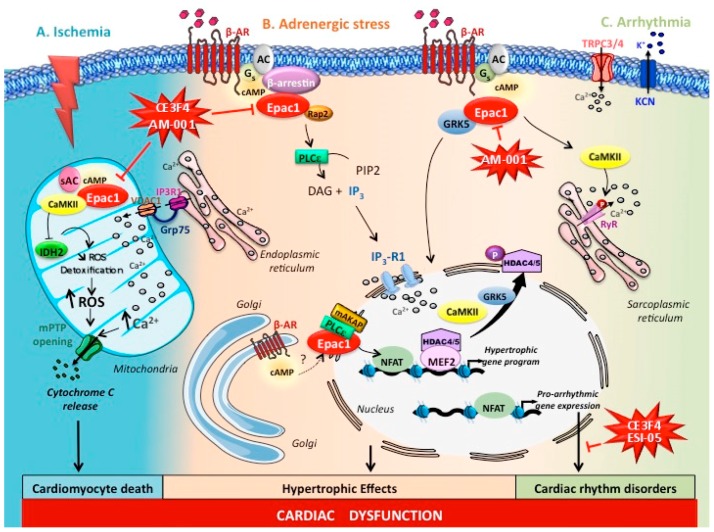
Epac1 signalosome in cardiac stress conditions. (**A**) Ischemia. In the context of cardiac ischemia, mitochondrial Epac1 is activated by the soluble adenylyl cyclases (sAC) and decreases reactive oxygen species (ROS) detoxification through isocitrate dehydrogenase 2 (IDH2) inhibition. In addition, Epac1 facilitates Ca^2+^ exchange from the endoplasmic reticulum (ER) to the mitochondrion by enhancing the formation of a Ca^2+^-handling macromolecular complex composed of voltage-dependent anion channel 1 (VDAC1), the chaperone glucose-regulated protein 75 (GRP75), and the inositol-1,4,5-trisphosphate (IP3) receptor 1 (IP3R1) between the ER and the mitochondrion interface. Epac1-induced Ca^2+^ overload and ROS accumulation promotes mitochondrial permeability transition pore (mPTP) opening and cardiomyocyte death. (**B**) Adrenergic stress. Under chronic β-adrenergic receptor (β-AR) stimulation, the β-arrestin/Epac1 complex is recruited at the plasma membrane and activates a Rap2/phospholipase C (PLC)ε pathway to induce 1,4,5-trisphosphate production. This signalling pathway converges to the nucleus to promote nuclear Ca^2+^ load, Ca^2+^/calmodulin-dependent protein kinase II (CaMKII) activation, histone deacetylase type 4 (HDAC4) nuclear export and eventually activation of the prohypertrophic transcription factor, myocyte enhancer factor 2 (MEF2). In addition, Epac1 induces G-protein receptor kinase 5 (GRK5) nuclear import and histone deacetylase type 5 (HDAC5) nuclear export to promote MEF2 activity. Yet, Epac1 prohypertrophic signalling includes the phosphatase calcineurin (CaN) and nuclear factor of activated T cells (NFAT). cAMP synthesized at the Golgi by intracellular β-AR may also stimulate the prohypertrophic Epac1/PLCε/A-kinase anchoring protein (mAKAP) signalling at the nuclear membrane of cardiomyocytes. (**C**) Arrhythmia. Epac1 activation contributes to cardiac rhythm disorders by increasing the expression level of pro-arrhythmic channels (transient receptor potential canonical (TRPC) 3/4 and potassium voltage-gated channel (KCN)). Epac1 also promotes spontaneous diastolic Ca^2+^ leak via CaMKII-dependent ryanodine receptors (RyR) hyperphosphorylation.

A key question is to determine the molecular mechanisms that link Epac1 to its myocardial detrimental effect in stressful situations. As for PKA, Epac proteins are expressed within different subcellular compartments including the nucleus and plasma membrane and coordinate discrete cellular responses in a spatiotemporally regulated fashion [7,9]. This is well illustrated in the context of cardiomyocyte hypertrophy. Compelling evidence indicated that the Ca^2+^/calmodulin-dependent protein kinase II (CaMKII) is a key effector of Epac1 hypertrophic signalling [9,52,60]. Specifically, Epac1 interacted with the scaffold protein β-arrestin, which is mandatory for Epac1 prohypertrophic action [61,62]. This signalling pathway converges to the nucleus where it induces the nuclear export of the histone deacetylase 4 (HDAC4) in a CaMKII dependent manner (Figure 2). HDACs are epigenetic enzymes that remove the acetyl groups from acetylated histones, increase chromatin condensation, and suppress gene transcription [63]. The Class IIa proteins, HDAC4 and HDAC5 function as negative regulators of cardiomyocyte hypertrophy [63]. Once phosphorylated by CaMKII, HDAC4 and HDAC5 are extruded out of the nucleus and subsequently relieve their inhibition on the prohypertrophic transcription factor myocyte enhancer factor 2 (MEF2) [62,64] (Figure 2). As expected, Epac1 activation increased MEF2 transcriptional activity in a CaMKII dependent manner [64].

Intriguingly, recent works suggested that intracellular β-ARs located at the Golgi could generate a specific pool of cAMP to stimulate a prohypertrophic Epac1/phospholipase C (PLC)ε signalling, which is scaffolded at the nuclear envelope of cardiomyocytes to the muscle-specific A-kinase anchoring (mAKAP) scaffolding protein [65,66] (Figure 2). Other studies demonstrated that an Epac1-PLC signalling could cause protein kinase D (PKD) activation and nuclear Ca^2+^ increase via the perinuclear inositol-1,4,5-trisphosphate (IP3) receptor resulting in the activation of CaMKII-dependent epigenetic regulation involving HDAC4 and HDAC5 [7,64,67,68]. Additional prohypertrophic Ca^2+^ signalling molecules that are modulated by Epac1 include transient receptor potential canonical (TRPC) channels that can initiate cardiomyocyte hypertrophy through increased Ca^2+^ influx and calcineurin/nuclear factor of activated T cells (NFAT) activity [9,69].

GRK2 and GRK5 are important regulators of G protein-coupled receptors (GPCRs) function and mediate the uncoupling and internalization of activated β-ARs in response to catecholamines [70]. In addition to this canonical GPCR desensitization, emerging evidence showed that GRKs are capable of regulating cardiac hypertrophic responses [70]. Of particular interest, Laudette and colleagues [45] recently found that Epac1 constitutively interacted with GRK2 and GRK5 in cardiac myocytes. Blocking Epac1 with the Epac1 inhibitor AM-001 prevented β-AR-induced Epac1/GRK5 complex formation, but not Epac1/GRK2 interaction indicating a specific role of Epac1 in GRK5 signalling. Importantly, the authors reported that Epac1 promoted the nuclear translocation of GRK5, which induced the nuclear export of HDAC5 and subsequent MEF2 prohypertrophic effect (Figure 2). Consistent with the nuclear/perinuclear localization of Epac1 [71], the aforementioned studies clearly indicate that Epac1 exerts via the non-canonical nuclear action of GRK5 epigenetic regulation to facilitate the development of cardiac hypertrophy. Further studies are required to elucidate other Epac1 epigenetic histone marks and Epac1 target genes that promote pathological cardiac remodelling. In addition, the role of GRK2 in Epac1 signalling deserves further investigation.

### 4.3. Role of Epac1 in Other Cardiac Disorders

#### 4.3.1. Atrial and Ventricular Arrhythmias

In addition to cardiac hypertrophy and HF, Epac1 pharmacological inhibitors might have other important clinical implications in cardiology. Indeed, various reports in the literature support the role of Epac1 in the development of the cardiac rhythm disorders, atrial and ventricular arrhythmias [9]. Studies performed in isolated adult rat ventricular myocytes showed that the preferential agonist Epac1, 8-pCPT-cAMP enhanced pro-arrhythmia mechanisms, including spontaneous diastolic Ca^2+^ leak via an hyperphosphorylation of the RyR and increased action potential duration [72,73] (Figure 2). Epac1 also influenced the expression level of proarrhythmic channels including the slow delayed-rectifier potassium K^+^-current (IKs) subunit potassium voltage-gated channel (KCN) and TRPC3/4 [69,74] (Figure 2). The arrhytmogenic effect of 8-pCPT-cAMP was confirmed in Langendorff-perfused and isolated murine hearts [75,76]. Yet, perfusion of isolated mouse failing hearts with 1 mM CE3F4 decreased the inducibility of atrial fibrillation induced by electrical stimulation [77]. Recent functional analyses of genetically engineered mouse models further demonstrated that Epac1 deficiency resulted in reduced susceptibility to atrial fibrillation (the most common sustained cardiac arrhythmia) and ventricular arrhythmia [58]. Consistently, the Epac1-selective inhibitor CE3F4 shortened the duration of the pacing-induced atrial fibrillation and reduced the incidence of sympathetic activation-induced ventricular arrhythmias, suggesting that Epac1 pharmacological inhibition was an effective strategy in the prevention of arrhythmia [78].

However, the role of Epac in promoting arrhythmias does not seem to be entirely devoted to the Epac1 isoform, at least in mouse. Indeed, Epac2 knockout mice were protected from β-AR–induced ventricular arrhythmia and showed reduced RyR-induced diastolic Ca^2+^ release [55]. This finding is consistent with the localization of Epac2 at T tubules, the site of RyR expression in cardiomyocytes [55]. Contrasting to these studies, an Epac2 pharmacological inhibitor, ESI-05 increased early after-depolarization arrhythmia and the frequency of spontaneous Ca^2+^ sparks in adult rat ventricular myocytes, suggesting that Epac2 inhibition may be proarrhythmic [79]. These results reveal the complexity of cAMP signalling and demonstrate the necessity to perform additional in vivo studies to determine the functional profile of cardiac Epac isoforms. Pharmacological studies in large animals are a prerequisite for validating the therapeutic potential of Epac1 inhibition in cardiac rhythm disorders.

#### 4.3.2. Cardiac Ischemia

Epac1 contains a functional mitochondrial targeting sequence raising the question of the biological action of mitochondrial Epac1 in cardiomyocytes [53,80]. In addition to ATP synthesis, mitochondria play a crucial role in Ca^2+^ buffering, reactive oxygen species (ROS) production, and apoptosis. This is well illustrated in the context of myocardial ischemia-reperfusion (I/R), a clinical relevant form of myocardial injury that occurs when the blood supply to the heart is interrupted (ischemia) and then re-established to return blood flow into the occluded coronary artery of the heart, a process defined as reperfusion [81]. I/R is the current therapeutic strategy for the management of acute MI since it reduces the myocardial infarct size and improves the clinical outcome of the patient. However, the process of reperfusion can itself induce burst of mitochondrial ROS and Ca^2+^ overload which stimulate the opening of the mitochondrial permeability transition pore (mPTP) leading to cytochrome C release, and induction of cardiomyocyte apoptosis [81] (Figure 2).

Ex vivo experiments performed in isolated heart rat showed that combined activation of PKA and Epac activation with cAMP analogues induced cardioprotection against I/R injury [82]. However, opposing results were obtained in vivo when Epac1 was specifically inhibited. Indeed, Fazal and colleagues [53] demonstrated that Epac1-deleted mice were protected against I/R injury by reducing infarct size and cardiomyocyte apoptosis. Most importantly, in a follow up study, the Epac1 inhibitor AM-001 directly phenocopied the genetic deletion of Epac1 in wild-type mice, suggesting that targeting Epac1 is an effective strategy to limit the lesion of I/R [45]. Mechanistically, Epac1 was activated by cAMP produced by sAC during ischemia, and promoted mitochondrial Ca^2+^ overload and ROS accumulation to trigger mPTP opening and cardiomyocyte death [53]. Although a previous study showed that the cAMP-Epac1 pathway could reduce mitochondrial Ca^2+^ entry in neonatal rat cardiomyocytes [83], further experiments performed in a more pathophysiological context, demonstrated that Epac1 facilitated Ca^2+^ exchange between the endoplasmic reticulum (ER) and the mitochondrion by promoting the formation of a Ca^2+^-handling macromolecular complex composed of voltage-dependent anion channel 1 (VDAC1), the chaperone glucose-regulated protein 75 (GRP75), and the IP3 receptor 1 (IP3R1) between the ER and the mitochondrion interface [53]. In addition, Epac1 inhibited in the mitochondrial matrix, the isocitrate dehydrogenase 2 (IDH2), a critical enzyme of the tricarboxylic acid cycle involved in ROS detoxification [53] (Figure 2). Taken together, these results show that Epac1 inhibition is cardioprotective. Intriguingly, Surinkaew and collaborators [84] reported that in mice, continuous infusion of the PDE resistant and Epac activator, Sp-8-pCPT-2’-O-Me-cAMPS improved post-MI induced remodelling and dysfunction. However, the non-selective Epac antagonist, ESI-09 did not influence infarct size in this model. Further studies employing a combination of genetic and pharmacological manipulations of Epac1 are required to further understand Epac1 role and signalling in the post-MI context.

## 5. Conclusions

Since the discovery of Epac pharmacological inhibitors, many Epac1 dependent biology processes have been successfully revealed [7]. Compelling evidence indicate that Epac1 forms discrete signalosomes with specific molecular partners inside distinct subcelullar compartments of cardiomyocytes. Epac1 activation dysregulates Ca^2+^ homeostasis and ROS production, and influences gene expression, contributing to the development of cardiac remodelling and diseases. Although some discrepancies exist in the literature that can be partially explained by the nature of the stress inducer, several recent reports combining both genetic and pharmacological manipulation of Epac1, provided proof-of-concept for the therapeutic effectiveness of inhibiting Epac1 activity in cardiac disease using small-molecule pharmacotherapy. Of particular importance, Epac1 inhibition does not alter basal cardiac function. Hence, the use of Epac1 inhibitory compounds may not display second side effects on cardiac function and their potential therapeutic application may also circumvent some of the undesirable effects of current treatment such as β-blockers. Since Epac1 seems to be mainly activated in stress conditions where cAMP concentration is high, uncompetitive and noncompetitive mode of Epac1 inhibition may represent a pharmacological advantage over competitive Epac1 inhibitors, which are mostly active at low agonist concentrations.

## Abbreviations

AA	Amino acid
β-AR	β-adrenergic receptor
cAMP	3′,5′-Cyclic adenosine monophosphate
sAC	soluble adenylyl cyclase
AKAPs	A-kinase anchoring proteins
ATP	adenosine triphosphate
CaMKII	Ca^2+^/calmodulin-dependent protein kinase II
CDC25-HD	cell division cycle 25 homology domain
CNBD	cyclic-nucleotide-binding domain
CNG	cyclic nucleotide gated
DEP	Dishevelled, Egl-10, Pleckstrin
Epac	exchange protein directly activated by cAMP
ER	endoplasmic reticulum
GEF	guanine-nucleotide-exchange factor
GPCRs	G protein-coupled receptors
GRK	G-protein receptor kinase
GRP75	chaperone glucose-regulated protein 75
HDAC	histone deacetylase
HF	heart failure
IDH2	isocitrate dehydrogenase 2
IK	potassium K+-current
IP3	inositol-1,4,5-trisphosphate
IP3R1	IP3 receptor 1 (IP3R1)
I/R	ischemia-reperfusion
MEF2	myocyte enhancer factor 2
MI	myocardial infarction
mPTP	mitochondrial permeability transition pore
NFAT	nuclear factor of activated T
PDE	phosphodiesterases
PKA	protein kinase A
PKD	protein kinase D
PKG	protein kinase G
PLC	phospholipase C
POPDC	popeye domain-containing proteins
RA	Ras-association domain
REM	Ras-exchange motif
RyR	ryanodine receptors
ROS	reactive oxygen species
SAR	structure-activity relationship
TRPC	transient Receptor Potential Canonical
VDAC1	voltage-dependent anion channel 1
